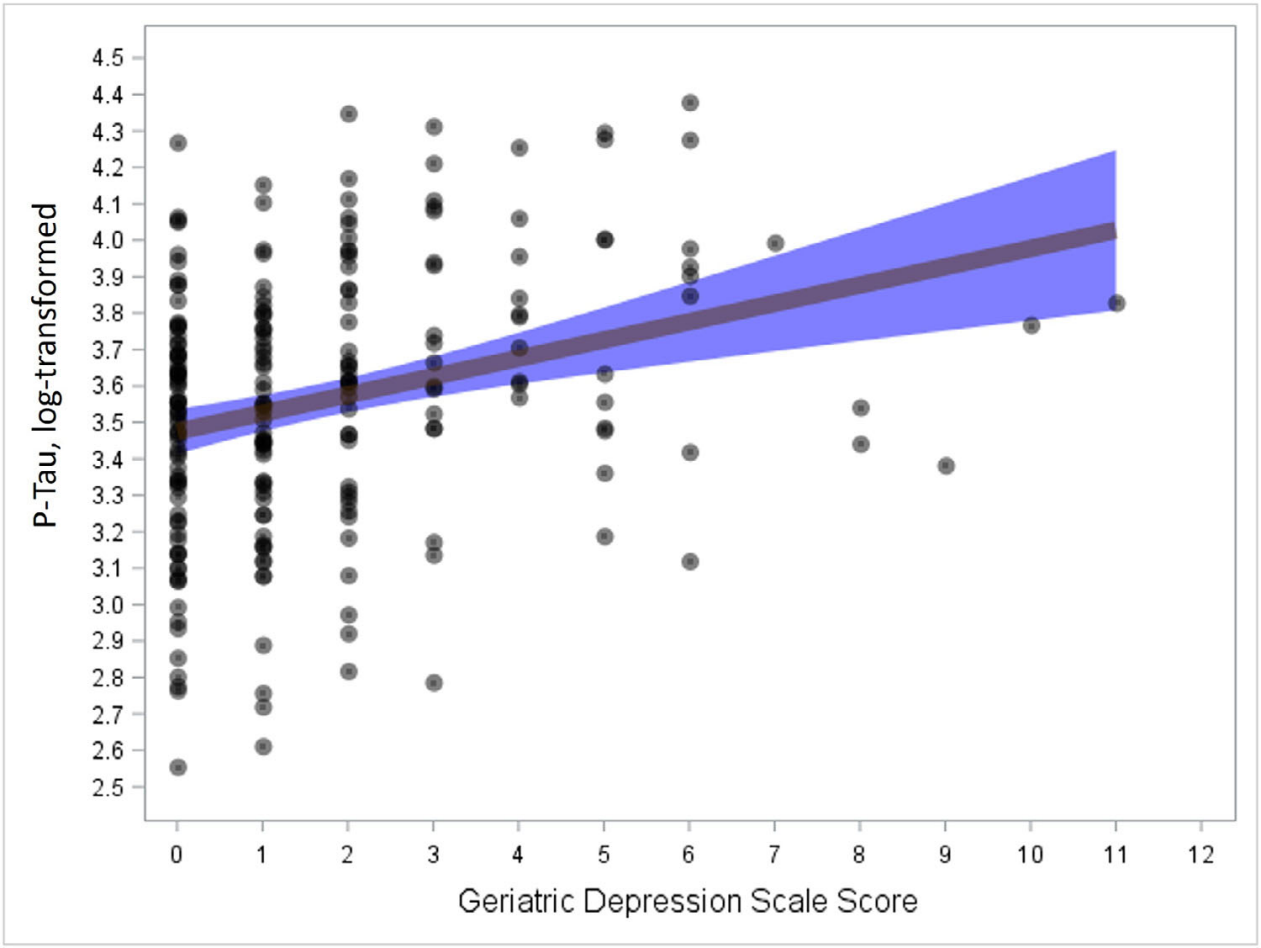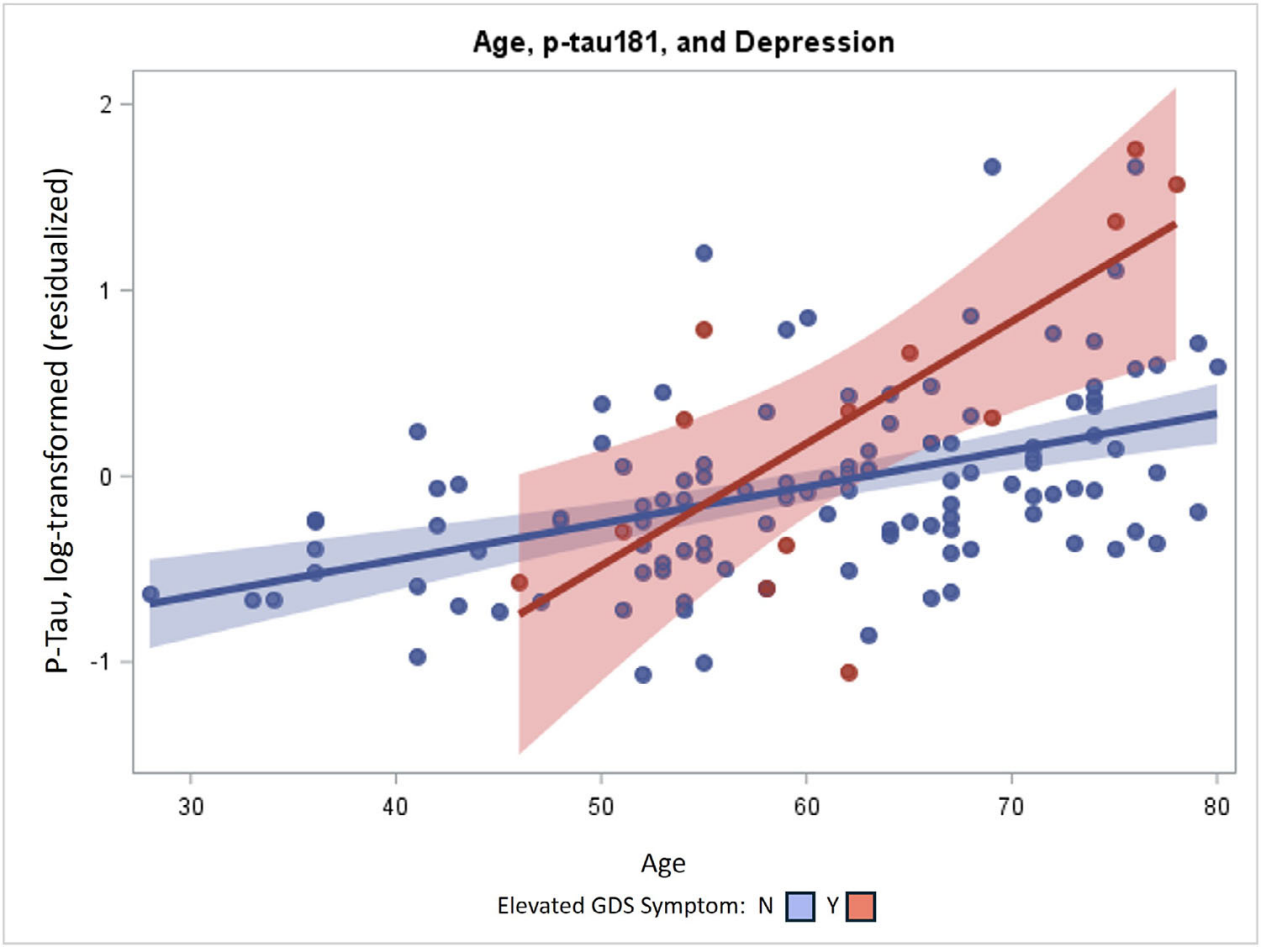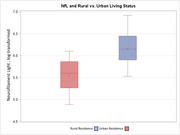# Psychosocial Functioning and Residential Status Associate with Cognition and Biomarkers for Alzheimer's Disease and Related Dementias across Adulthood

**DOI:** 10.1002/alz70856_098671

**Published:** 2025-12-24

**Authors:** Patrick J Smith, Kim G Johnson, Heidi L Roth, Guy G Potter, Sara Patillo, Weili J Lin, Allen J Song, Miles Berger, Richard J O'Brien, Andy Liu, Michael W Lutz, Sheng Luo, Andrea Bozoki, Kathleen A. Welsh‐Bohmer, Gwenn A Garden, Heather Whitson

**Affiliations:** ^1^ University of North Carolina at Chapel Hill, Chapel Hill, NC, USA; ^2^ Duke University, Durham, NC, USA; ^3^ UNC, Chapel Hill, NC, USA; ^4^ Duke University– Joseph and Kathleen Bryan Alzheimer's Disease Research Center, Durham, NC, USA; ^5^ Duke University Medical Center, Durham, NC, USA; ^6^ Duke/UNC Alzheimer's Disease Research Center, Duke University School of Medicine, Durham, NC, USA; ^7^ Duke University School of Medicine, Durham, NC, USA; ^8^ Duke Department of Neurology, Durham, NC, USA; ^9^ University of North Carolina, Chapel Hill, NC, USA

## Abstract

**Background:**

Psychosocial factors, including mood function and residential status (urban vs. rural), have been suggested to affect the risk of Alzheimer's Disease and Related Dementias (ADRD). Few studies, however, have attempted to examine the associations between these psychosocial factors and ADRD cerebrospinal fluid (CSF) biomarkers, which provide unique information on prodromal ADRD risk prior to the onset of clinical impairments in neuropsychological function.

**Method:**

We examined the associations between psychosocial functioning with cognitive function and ADRD biomarker profiles among young, middle‐aged, and older adults participating in the Duke‐UNC ADRC cohort. Among the 243 enrolled participants, 162 underwent CSF biomarker assessments. Depressive symptoms were assessed using the Geriatric Depression Scale (GDS). Residential status was indexed as either Urban or Rural using the 2010 United States urban maps from ArcGIS. CSF markers included Aβ42/40, phosphorylated tau (*p*‐tau181) and neurofilament light (NfL). Cognitive function was assessed from the National Alzheimer's Coordinating Center Uniform Data Set. Factor analysis was used to aggregate individual cognitive subtests into two unit‐weighted cognitive domain scores: Executive, Language, and Visuospatial Function (ELVF) and Memory. General linear models were used to examine the associations between psychosocial functioning with cognitive function and ADRD biomarkers, controlling for age, education, gender, race, comorbidities, and APOE genotype.

**Result:**

Participants ranged from 28 to 80 years of age (mean = 59.8 years [SD = 11]), were mostly female (69%) and white (20%), and nearly half had ≥ one APOE risk allele. Levels of depressive symptoms were minimal, with only 27 (11%) exhibiting clinically elevated levels. The majority of participants resided in urban areas (74%). Greater depressive symptoms associated with worse Memory performance (*p* = .005) and higher *p*‐tau181 levels (*p* = .026; Figure 1). The association between depressive symptoms and *p*‐tau181 also varied by age (*p* = .034 for interaction), such that the age and *p*‐tau181 association was stronger in the presence of elevated depressive symptoms (Figure 2). Individuals who resided in rural vs. urban areas showed lower ELVF performance (*p* = .033) and higher NfL levels (*p* = .048; Figure 3).

**Conclusion:**

Psychosocial characteristics associate with cognitive function and ADRD biomarkers among middle‐aged adults.